# Effect on alpha-amylase Kinhibitor genetically modified (GM) pea consumption on lung inflammation in a mouse model of allergic asthma

**DOI:** 10.1186/2045-7022-1-S1-O14

**Published:** 2011-08-12

**Authors:** Rui-Yun Lee, Daniela Reiner, TJV Higgins

**Affiliations:** 1 Medical University of Vienna, Division of Immunology, Allergy and Infectious Diseases, Experimental Allergy, Department of Dermatology, Vienna, Austria; 2 Commonwealth Scientific and Industrial Research Organization Plant Industry, Canberra, ACT, Australia

## 

Transgenic field peas (pisum sativum) expressing the alpha-amylase inhibitor (AAI) protein normally found in common beans (Tendergreen bean) are completely protected from infestation with pea weevils tested in the laboratory, greenhouse and field over several generations, and at many sites for efficacy against the insect pests. Previously, transgenic AAI pea feeding in mice was shown to influence allergic responses to an unrelated, non-crossreactive allergen compared to pinto bean-fed mice. To determine whether this is a universal finding, we used a different protocol in which we injected female BALB/c mice on days 0 and 21 with ovalbumin (OVA) intraperitoneally and nebulized them with OVA on days 28 and 29 to initiate allergic asthma and then allowed mice to recover until they were re-exposed to OVA for the induction of a disease exacerbation. Our aim was to test the effect of gavage feeding AAI peas (100 mg/ml), Tendergreen bean, or PBS two times a week for 4 weeks prior to inducing disease or inducing disease exacerbation. We detected no differences in lung and airway inflammation, mucus hypersecretion and allergen-specific antibody in AAI-pea fed- compared to isogenic pea-fed- mice at the onset of allergic asthma or at the time of disease exacerbation. However, in acute onset disease, pea fed mice had more severe lung inflammation and elevated mucus hypersecretion compared to Tendergreen bean-fed mice. Taken together, our data suggest that factors in peas other than the transgene appear to have an adjuvant effect on allergic responses to other unrelated allergens.

